# Cervical Cancer Awareness among Women in Tanzania: An Analysis of Data from the 2011-12 Tanzania HIV and Malaria Indicators Survey

**DOI:** 10.1155/2018/2458232

**Published:** 2018-05-02

**Authors:** Fabiola V. Moshi, Elisa B. Vandervort, Stephen M. Kibusi

**Affiliations:** College of Health Sciences, School of Nursing and Public Health, University of Dodoma, Dodoma, Tanzania

## Abstract

**Background:**

Awareness about cervical cancer is a first step in the process of screening and early treatment. The purpose of this study was to provide better understanding of basic knowledge about cervical cancer among women of reproductive age in Tanzania.

**Method:**

Data were analyzed from the 2011-2012 Tanzania HIV and Malaria Indicators Survey (THMIS) and a sample of 5542 sexually active women from 15 to 49 years of age were included in the analysis.

**Results:**

Overall knowledge about cervical cancer was high among interviewed women. Only 30.9% of women had never heard about cervical cancer. The predictors of awareness were having secondary or more level of education (AOR = 3.257, 95% CI 2.328–4.557, *p* < 0.001), residing in urban (AOR = 1.365, 95% CI 1.093–1.705, *p* < 0.01), being affluent (AOR = 2.685, 95% CI 2.009–3.587, *p* < 0.001), having one to four children (AOR = 1.36, 95% CI 1.032–1.793), and age of 30–34 years (AOR = 3.15, 95% CI 2.353–4.220, *p* < 0.001), 35–39 years (AOR = 2.46, 95% CI 1.831–3.308, *p* < 0.001), and 40–44 years (AOR = 3.46, 95% CI 2.497–4.784, *p* < 0.001).

**Conclusion:**

While the cervical cancer landscape in Tanzania has evolved since this survey, coverage has not yet been achieved and access to cervical cancer prevention services for rural women and girls remains a concern. Women who were least likely to be aware of cervical cancer were rural women, less affluent women, those with limited education, and those with limited access to the formal economy. Arguably, these are the women who are most at risk for cervical cancer. To close this gap, Tanzania's ongoing efforts to increase access to high-quality cervical cancer prevention services for all women at risk are commendable.

## 1. Introduction

Cervical cancer is caused by the human papilloma virus (HPV), the most common viral infection of the reproductive tract. Almost all sexually active individuals will become infected with HPV at some point in their lives, and some may repeatedly be infected. The peak time for infection is shortly after becoming sexually active, and most individuals with healthy immune systems will clear the virus within a few years [1]. Prolonged infection with high-risk oncogenic types of HPV puts women at risk for development of cervical cancer. To mitigate this risk, primary and secondary prevention strategies have been implemented in the global north, dramatically reducing the rate of cervical cancer diagnosis and death [1, 2].

In the global south, cervical cancer remains the second most common cancer (after breast cancer) among women of reproductive age. Worldwide, it is the third most common carcinoma after breast cancer and colorectal cancer [1, 3]. Unlike other cancers, cervical cancer is almost 100% preventable by ensuring that women receive quality screening and treatment of precancerous lesions. The impact of secondary prevention efforts for cervical cancer prevention in high resource settings is impressive [3]. Despite this, the World Health Organization (WHO) estimates of global cervical cancer rates remain sobering, with more than 500,000 new cases diagnosed each year. Of those cases, more than 85% of women hail from developing countries where access to primary and secondary prevention is far from universal [3, 4]. In these settings, most women diagnosed with cervical cancer in developing countries present at late stages when curative treatments are often no longer possible [1].

East Africa has the highest rate of cervical cancer in the world. In this region, the age-standardized incidence rate (ASR) is estimated at 42.7 new cases per 100,000 women [3]. ASR rates range from Malawi at 75.9/100,000 to Kenya at 40.1/100.000 [3]. Tanzania ranks second in the region with an ASR of 54.9/100,000 women [3]. Similar to other East African countries, cervical cancer is the leading cause of cancer and cancer-related death among Tanzanian women. Each year more than 7,300 Tanzanian women are diagnosed with cervical cancer [3]. More than half of these women die as they are diagnosed at a late stage of the disease [3].

The variation in cervical cancer rates by region mirrors access to primary and secondary cervical cancer prevention. Given the tremendous burden in Sub-Saharan Africa, the impact of HIV cannot be overlooked. It is well known that women with HIV are at unique risk for persistent HPV infection and cervical dysplasia. As such, women with HIV in Sub-Saharan Africa may have access to effective treatments for HIV but lack access to life-saving screening and treatment for precancer of the cervix [1, 5].

Effective screening and treatment for precancer of the cervix are a secondary prevention strategy that has been implemented globally to prevent cervical cancer. Commonly used screening tests include cytology (Pap), HPV testing, and visual inspection with acetic acid (VIA). Myriad studies have examined and reexamined the risks and benefits of each screening approach [1, 6, 7]. Regardless, highly effective modalities for screening and treatment of cervical precancer are widely available in the global north to prevent cervical cancer. For most women in the global south, universal access to high-quality cost-effective screening and treatment remains a distant goal and access to HPV vaccines for primary prevention also remains unequal [1].

Similar to women in other developing countries, most Tanzanian women with cervical cancer are diagnosed at late stages when curative treatments are no longer possible. Since 2002, the Tanzanian Ministry of Health and Social Welfare (MoHSW) has collaborated with the WHO, the International Agency for Research on Cancer (IARC), the international nonprofit Jhpiego, and numerous other local and international NGO partners to scale up cervical cancer prevention efforts for Tanzanian women [8, 9]. In accordance with the MoHSW's Service Delivery Guidelines for Cervical Cancer Prevention Services, the use of visual inspection with acetic acid (VIA) and cryotherapy is the secondary prevention approach that has been widely implemented in Tanzania [8]. This secondary prevention strategy is well supported by the research as a cost-effective strategy for cervical cancer prevention [1, 7] and is a recommended strategy by the WHO for cervical cancer prevention in low resource settings [1].

At a fundamental level, cervical cancer prevention programming focuses on two main areas: (1) the supply of prevention services on the part of health systems and (2) generating demand for services at the community level. Knowledge about an illness does not necessarily translate into a demand for services, but it is a first step in the process [1]. To better understand baseline community knowledge about cervical cancer in Tanzania at the time when the MoHSW was scaling prevention efforts, we reviewed data from the 2011-2012 Tanzania HIV and Malaria Indicators Survey.

## 2. Materials and Methods

### 2.1. The Study Area and Period

The study was conducted among women of reproductive age, aged 15–49 years living in Tanzania sampled from a population of 10,905,117 women accounting for 47.3% of women in Tanzania [10].

### 2.2. Design

A nationally based cross-sectional survey of a representative sample of individuals aged 15–49 years living in Tanzania was conducted in the period between December 2011 and May 2012.

### 2.3. Data Sources

The source of data was the 2011-2012 Tanzania HIV and Malaria Indicators Survey (THMIS). The survey was conducted by the National Bureau of Statistics (NBS) in collaboration with the Tanzania Commission for AIDS (TACAIDS) and the Zanzibar AIDS Commission (ZAC), the Ministry of Health and Social Welfare (MoHSW), and the USAID-funded Measure DHS project from December 16, 2011, to May 24, 2012.

### 2.4. Sampling Technique and Sample Size

The 2002 Population and Housing Census (PHC) which covered all of the 30 regions of Tanzania was used as a sampling frame. The first stage involved selecting sample points (clusters) consisting of enumeration areas (EAs) delineated for the 2002 PHC. A total of 583 clusters were selected. On Tanzania's mainland, 30 sample points were selected in Dar es Salaam. Additional 20 data points were selected in each one of the other 24 regions. In Zanzibar, 15 sample points were selected in each of Zanzibar's five regions. The second stage of selection involved systemic sampling of households. Prior to fieldwork, a household listing operation was undertaken in all the selected areas. From these lists, households to be included in the survey were then selected. A total sample size of 10,496 households was identified through the following process: approximately 18 households were selected from each sample point thereby determining the total. Weighting factors were utilized to obtain results proportional at the national level. Eligible respondents included all women and men aged 15–49 who either permanently live in the selected household or were visitors who stayed in the home on the night prior to the survey. In total, 10,067 women completed interviews, with a response rate of 96%. The main reason for nonresponse among eligible women was the failure to find them at home despite repeated visits to the households.

The question regarding cervical cancer awareness was given to women of reproductive age of 15–49 years. Of the 10,967 women who completed interviews, 9693 responded to the question regarding cervical cancer awareness. From this sample, 8365 participants were further selected for being sexually active. Participants with missing data on important covariates particularly regarding assertive safe sexual behavior were excluded, remaining with a final sample of 5542 women included in this analysis

### 2.5. Data Collection Tools

The 2011-12 THMIS utilized two questionnaires: a Household Questionnaire and an Individual Questionnaire. These questionnaires were based on the Measure DHS standard AIDS Indicator Survey and Malaria Indicator Survey questionnaires. The questionnaires were adapted to reflect Tanzanian population and relevant health issues of Tanzania. Following adaption, the questionnaires were translated into Kiswahili, Tanzania's national language. The data presented in this study is based on the individual questionnaire.

### 2.6. Variables

Guided by literature review, key variables were identified from the 2011-12 THMIS individual questionnaires. A conceptual framework was developed comprising a list of primary independent variables (such as socioeconomic and demographic variables), intermediate independent variables (such as number and timing of antenatal visits), and the outcome variable, cervical cancer awareness.

### 2.7. Outcome Measures

The outcome was an awareness about cervical cancer (which was a dichotomous variable) whereby a participant was or was not aware of cervical cancer.

### 2.8. Independent Variables

The socioeconomic and demographic variables that have been theoretically and empirically reported to influence women's awareness of health issues were included in this study. Demographic characteristics included variables such as age grouping by 5-year intervals, the location of residence (rural or urban), education level, a woman's marital status, and occupation status. Other sociodemographic characteristics included parity, Tanzanian zone of residence, and wealth index categorized as poorest, poorer, middle income, richer, and richest. The survey also included intermediate factors potentially related to cervical cancer awareness. These included a woman's assertiveness regarding safe sexual behavior, access to reproductive health services, and indicators for sexually transmitted infections.

### 2.9. Statistical Analyses

Descriptive statistics for sample's sociodemographic characteristics and cervical cancer awareness were calculated. Sociodemographic differences in cervical cancer awareness were assessed by chi-square testing. For all the analyses, the level of significance was set at *p* < 0.05 (2-tailed). To generate crude (OR) and adjusted odds ratios (AOR) both bivariate and multiple logistic regression were employed. Odds ratios were estimated to determine the strength of the associations. Confidence intervals (CIs) of 95% were used for significance testing. Covariates were simultaneously entered into the multiple regression models. Analyses were performed using SPSS version 16. To allow for adjustments for the cluster sampling design and sampling probabilities across clusters and strata, sample weighting was applied.

### 2.10. Ethical Considerations

The procedures for the THMIS data collection and the survey content and protocol were approved by Tanzania's National Institute for Medical Research (NIMR), the Zanzibar Medical Ethics and Research Committee (ZAMREC), the Institutional Review Board of ICF International, and the Centers for Disease Control and Prevention in Atlanta, USA. After being read a document emphasizing the voluntary nature of the survey, participants provided verbal informed consent. The household interviews took place under the most private conditions afforded by the environments encountered. If privacy could not be insured, the interviewers were instructed to skip the module.

## 3. Results and Discussion

### 3.1. Results

Table 1 shows the sociodemographic characteristics of participants and their awareness about cervical cancer. Most of the participants had completed at least primary education. There was a significant relationship (*p* < 0.001) between education level and awareness about cervical cancer. Among women without education, 44.3% had never heard of cervical cancer. Young respondents (ages 15 to 19 years) had the highest percentage of women (50.2%) who had never heard about cervical cancer (*p* < 0.001). There was also a statistically significant relationship (*p* < 0.001) between the women's occupation and awareness about cervical cancer. Among self-employed women, 32.9% were not aware of cervical cancer.

Table 2 shows the distribution of participants by potential factors which may affect awareness about cervical cancer. When analyzed by income, the most affluent group had the highest awareness about cervical cancer (87.1%). This was contrasted by a much lower awareness among less affluent women. Among those women, only 57.4% had ever heard about cervical cancer (*p* < 0.001). When comparing different zones of Tanzania, women from Eastern Tanzania had the highest percentage (85%) of women who had heard about cervical cancer, while women from the South Western Highlands had the lowest percentage (59.9%) of women who had ever heard about cervical cancer (*p* < 0.001). Awareness about cervical cancer was also compared to parity (reported number of children delivered). Respondents with no children had the lowest percentage (58.6%) of awareness about cervical cancer and women who had one to four children had the highest awareness (71.4%) about cervical cancer (*p* < 0.001). When considering the place of residence, women from urban areas had the highest percentage (84.6%) of knowledge about cervical cancer while among women, only 65% had ever heard about cervical cancer (*p* < 0.001).

The results of the logistic regression analysis are presented in Table 3. This analysis demonstrated that a woman was aware of cervical cancer if she had the following characteristics: aged between 30 and 49 years, more affluent, having had completed at least secondary school, living in an urban area, and self-employed. Interestingly, employed women were less (4%) likely to have had heard about cervical cancer compared to unemployed women.

## 4. Discussion

Through this review of the data, a profile emerges of a Tanzanian woman most likely to know about cervical cancer in 2011-2012. She was more likely to live in an urban setting, be older than 30 years of age, have completed secondary education, be self-employed, and be affluent. In contrast, those most at risk for lack of awareness about cervical cancer were rural Tanzanian women with limited education and limited access to the formal economy and women of young reproductive age. Despite the scaling up of secondary cervical cancer prevention efforts with VIA and cryotherapy in Tanzania since 2011-2012, the availability of high-quality cervical cancer prevention services for rural populations is uncertain. Even today, one wonders about the availability of screening and treatment for cervical precancer for rural women who know about the service availability and somehow manage to surmount the many barriers to accessing services [11, 12]. Despite impressive local efforts to scale up effective cervical cancer prevention services since 2011-2012, it is likely that not all Tanzanian women know about their risk for cervical cancer or where to access screening services. As such not all women in Tanzania have equal access to services [11, 12].

This analysis found that the vast majority of study participants (69%) had heard about cervical cancer as of 2011-2012. A similar study performed in Kilimanjaro Region among rural and urban women reported that the majority of women were knowledgeable about cervical cancer [11]. Given the high rates of cervical cancer in Tanzania, this knowledge is not surprising. Rural and urban Tanzanians have significant experience caring for family and community members with end-stage cervical cancer. It is possible that cervical cancer awareness was underreported in this 2011-2012 survey as less educated and less affluent Tanzanians may not have been familiar with the medical terminology used by the interviewers.

Rural women who are more apt to be poor and less educated were less knowledgeable about cervical cancer (65%), but overall knowledge was quite high in this survey. Knowledge among urban women (84.6%) mirrors findings from a Kenyan study where 87% of urban women were aware of cervical cancer [13]. This current analysis also found that the level of education of the respondents influenced their awareness of cervical cancer. With increasing education, a woman was more likely to be aware. This finding is consistent with other studies demonstrating cervical cancer knowledge correlating with higher education [14].

Our analysis of the 2011-2012 THMIS data found that the vast majority of women knew something about cervical cancer and that basic knowledge also increased as women aged. This has been demonstrated in other settings [15]. Unfortunately, this knowledge may be more linked with personal experience rather than effective community health education and personal experience with cervical cancer screening services. As the leading cause of cancer and cancer death among women ages 15 to 44 years of age in Tanzania, it is common for Tanzanian men and women to have a personal connection with someone who has had cervical cancer.

As such, in this setting there is a relatively robust awareness of the existence of cervical cancer among women of reproductive age. They know that this illness exists but commonly lack knowledge about the availability of screening and treatment services [11]. To increase screening and treatment coverage for cervical precancer, we must devise strategies to deepen knowledge at the community level and drive a demand for effective primary and secondary prevention services. By increasing core knowledge about cervical cancer risk and available prevention services, it may be possible to cultivate a demand for screening services for all women at risk.

Some programs have focused on increasing community awareness about signs and symptoms of cancer to strengthen a person's ability to detect early signs and symptoms of cancer. Studies have reported that knowledge and understanding of cancer risk factors and outcomes of cancer treatments influenced individual's intentions and participation in cancer prevention programs [16, 17]. In the case of cervical cancer, the early stages of cervical cancer are commonly silent, without symptoms. As such, women presenting with symptoms of cervical cancer usually have late-stage disease, and curative treatments are unlikely [1].

Luckily, cervical cancer tends to have a relatively long precancer stage, providing a convenient window for preventive screening and treatment for precancer. To prevent progression to cancer, women require access to early detection and treatment of precancerous lesions [1]. In Tanzania, women and men know that cervical cancer exists. But further intensive outreach and education are needed at the community level to deepen knowledge about prevention of cervical cancer and meaningfully connect women and girls with primary and secondary prevention services. A crucial layer of this process is for an individual to possess knowledge and understanding about woman's risk for cervical cancer and the local options available for prevention. If such information is not widely available and community structures are not engaged to assist with reproductive health education and mobilization, it understandably increases the risk that women will not receive quality screening and treatment, even if such is available.

The reasons for the high cervical cancer mortality burden in East Africa and Tanzania are complex. Regardless, it is evident that to reduce cervical cancer incidence in Tanzania and beyond, creative strategies must continue to be implemented to increase coverage of preventive services for cervical cancer. There are complex factors that contribute to Tanzania's continued high rates of death from cervical cancer. Simply put, if at least 80% of women had access to quality screening and treatment for precancer at least once in their life (when most at risk), cervical cancer rates in Tanzania could plunge rapidly [7]. With more than 5 million Tanzanian women between 30 and 50 years of age [18], there is a vast population to reach with cervical cancer screening services. Given the current health policy and funding climate, it is likely unwise for women of Tanzania to patiently wait for the health system and NGO partners to provide optimal screening and treatment services, especially for rural populations. It may be that cultivating a grassroots demand for services becomes a more effective way to influence the supply of primary and secondary cervical cancer prevention services. By driving demand at a local level, the health system may be encouraged to strengthen and respond.

To that end, continued engagement at the community level should engender creative and sustainable ways to increase knowledge and awareness about cervical cancer to drive a demand for effective primary and secondary prevention services. As demonstrated by the 2011-2012 THMIS data and numerous other studies in Tanzania [11, 12], women know about cervical cancer but often do not realize that there are effective preventive services available. To close this gap, we need creative multisectoral collaborations that will prioritize investments in effective reproductive health services for all women at risk in the global south, including a focus on primary and secondary cervical cancer prevention.

This study has some limitations, as it utilized data that is now relatively old. Knowledge today is likely increased given MoHSW and NGO partners' secondary prevention achievements, especially in communities where screening access and uptake have been high. Regardless, it is interesting to such a high baseline level of awareness at a time when MoHSW had really just begun to scale up cervical cancer prevention efforts.

## 5. Conclusion

This analysis looked at the 2011-2012 THMIS data to better understand cervical cancer knowledge among Tanzanian women at a time when the MoHSW was scaling up cervical cancer prevention efforts. While the cervical cancer landscape in Tanzania has evolved since this survey, coverage has not yet been achieved, and access to cervical cancer prevention services especially for rural women and girls remains a concern. Overall knowledge about cervical cancer was quite high among surveyed women, most likely due to personal experiences caring for someone with cervical cancer. Women who were least likely to be aware of cervical cancer were rural women, less affluent women, those with limited education, and those with limited access to the formal economy. Arguably, these are the women who are most at risk for cervical cancer. Since this survey, impressive gains have been made in the access to screening and treatment for precancer in Tanzania through the efforts of the MoHSW and NGO partners. Meanwhile, the vast majority of women at risk have not yet been screened, such women may not know of available screening services, and most Tanzanian girls have not yet received the HPV vaccine. To close this gap, Tanzania's efforts to increase access to high-quality cervical cancer prevention services for all women at risk are commendable. These efforts deserve continued local and international support to reduce unnecessary deaths from cervical cancer. Increasing demand at the community level for such services will continue to be a crucial component of the equation, especially among rural populations.

## Figures and Tables

**Table 1:** The distribution by sociodemographic characteristics and awareness about cervical cancer.

Variables	Awareness about cervical cancer				*p* value
	Not aware		Aware		
	*n*	%	*n*	%	
*Education:*					
No education	528	44.3	663	55.7	
Primary incomplete	250	35.8	448	64.2	*∗∗∗*
Primary completed	867	27.3	2307	72.7	
Secondary or more	67	14	413	86	
*Age groups: *					
15–19	218	50.2	216	49.8	
0–24	334	35	619	65	
25–29	374	32	794	68	*∗∗∗*
30–34	224	23.5	731	76.5	
35–39	287	30.7	647	69.3	
40–44	155	24.6	475	75.4	
45–49	119	25.4	350	74.6	
*Occupation: *					
Unemployed	162	27.3	432	72.7	
Self-employed	1494	32.9	3050	67.9	*∗∗∗*
Employed	56	13.8	349	86.2	

Here *∗∗∗* indicates *p* < 0.001. *N* = 5543; 2 participants were missing some information on independent variables: 2 on occupation.

**Table 2:** Distribution of participants by awareness about cervical cancer and the potential factors affecting awareness (chi-square).

Variables	Awareness about cervical cancer				*p* value
	Not aware		Aware		
	*n*	%	*n*	%	
*Wealth index: *					
Poorest	441	42.6	595	57.4	
Poorer	433	37.8	712	62.2	*∗∗∗*
Middle	378	34.6	713	65.4	
Richer	318	27.3	847	72.7	
Richest	142	12.9	962	87.1	
*Zones: *					
Eastern	90	15	510	85	
Western	138	31.6	299	68.4	
Southern	112	36.1	198	63.9	*∗∗∗*
Southern Highlands	244	38.7	386	61.3	
SW Highlands	240	40.1	359	59.9	
Central	129	20.1	513	79.9	
Northern	188	30.2	434	69.8	
Lake	507	33.1	1025	66.9	
Zanzibar	64	37.4	107	62.6	
*Parity: *					
0	146	41.4	207	58.6	
1–4	933	28.6	2332	71.4	*∗∗∗*
>4	633	32.9	1292	67.1	
*Place of Residence:*					
Urban	180	15.5	989	84.6	*∗∗∗*
Rural	1532	35	2843	65	

Here *∗∗∗* indicates *p* < 0.001.

**Table 3:** Adjusted odds ratios (AOR) for factors associated with awareness about cervical cancer among women of reproductive age (15–49 yrs) in Tanzania 2011-2012 (*N* = 5543).

Variable	AOR	95% CI	*p* value
*Age groups: *			
15–19	1		
20–24	1.58	1.212–2.054	*∗∗∗*
25–29	1.80	1.377–2.348	*∗∗∗*
30–34	3.15	2.353–4.220	*∗∗∗*
35–39	2.46	1.831–3.308	*∗∗∗*
40–44	3.46	2.497–4.784	*∗∗∗*
45–49	1.35	2.370–4.733	*∗∗∗*
*Zones: *			
Eastern	1		
Western	0.75	0.543–1.044	
Southern	0.49	0.345–0.691	*∗∗∗*
Southern Highlands	0.40	0.297–0.543	*∗∗∗*
SW Highlands	0.47	0.343–0.629	*∗∗∗*
Central	1.43	1.036–1.971	*∗*
Northern	0.61	0.452–0.830	*∗∗*
Lake	0.69	0.527–0.909	*∗∗*
Zanzibar	0.21	0.133–0.318	*∗∗* ∗
*Parity: *			
No child	1		
One to four children	1.36	1.032–1.793	*∗*
More than four children	0.95	0.694–1.301	
*Employment status:*			
Unemployed	1		
Employed	0.96	0.758–1.205	
Self-employed	1.51	1.091–2.085	*∗*
*Wealth: *			
Poorest	1		
Poorer	1.237	1.032–1.482	*∗*
Middle	1.383	1.147–1.668	*∗∗*
Richer	1.706	1.396–2.084	*∗∗∗*
Richest	2.685	2.009–3.587	*∗∗∗*
*Level of education: *			
No education	1		
Primary incomplete	1.434	1.17–1.758	*∗∗*
Primary completed	1.704	1.463–1.985	*∗∗∗*
Secondary or more	3.257	2.328–4.557	*∗∗∗*
*Area of residence: *			
Rural	1		
Urban	1.365	1.093–1.705	*∗∗*

Here *∗*, *∗∗*, and *∗∗∗* indicate *p* < 0.05, *p* < 0.01, and *p* < 0.001, respectively.

## Data Availability

The data that support this analysis are available from the 2011-12 Tanzania HIV and Malaria Indicators Survey (THMIS). This survey was conducted by the National Bureau of Statistics (NBS) in collaboration with the Tanzania Commission for AIDS (TACAIDS) and the Zanzibar AIDS Commission (ZAC), the Ministry of Health and Social Welfare (MoHSW), and the USAID-Funded Measure DHS project from December 16, 2011, to May 24, 2012. Data is available from the authors upon reasonable request and with permission from MEASURE DHS.

## Abbreviations

ASR: Age-standardized incidence rate
HIV: Human immunodeficiency virus
HPV: Human papilloma virus
MoHSW: Ministry of Health and Social Welfare
NBS: National Bureau of Statistics
NGO: Non-Governmental Organization
TACAIDS: Tanzania Commission for AIDS
THMIS: Tanzania HIV and Malaria Indicators Survey
VIA: Visual inspection with acetic acid
WHO: World Health Organization
ZAC: Zanzibar AIDS Commission.
